# Obesity and physical inactivity are associated with increased risks of cardiac conduction disease: a report from the Kailuan Cohort Study

**DOI:** 10.1038/s44325-024-00008-8

**Published:** 2024-07-25

**Authors:** Hongmin Liu, Xinmu Li, Peipei Liu, Haiyan Zhao, Liming Lin, Gary Tse, Jeffrey Shi Kai Chan, Gregory Y. H. Lip, Shouling Wu, Tong Liu

**Affiliations:** 1 Tianjin Key Laboratory of Ionic-Molecular Function of Cardiovascular Disease, Department of Cardiology, Tianjin Institute of Cardiology, Second Hospital of Tianjin Medical University, Tianjin, China; 2 Department of Cardiology, Kailuan General Hospital, Tangshan, China; 3 School of Public Health, North China University of Science and Technology, Tangshan, China; 4 School of Nursing and Health Studies, Hong Kong Metropolitan University, Hong Kong, China; 5 Cardiac Electrophysiology Unit, Cardiovascular Analytics Group, PowerHealth Limited, Hong Kong, China; 6 Liverpool Centre for Cardiovascular Science at University of Liverpool, Liverpool John Moores University and Liverpool Heart & Chest Hospital, Liverpool, United Kingdom; 7 Danish Center for Health Services Research, Department of Clinical Medicine, Aalborg University, Aalborg, Denmark

**Keywords:** Cardiology, Health care, Diseases, Cardiovascular diseases

## Abstract

Physical activity (PA) and obesity may alter the risks of cardiac conduction disease. Participants from the Kailuan cohort, who were free of cardiac conduction disease and with repeated measurements of electrocardiogram from 2006 to 2019, were included. The primary outcome was cardiac conduction disease. The secondary outcomes were atrioventricular block and intraventricular block. Cox regression was used to assess the association between obesity, PA, and the risks of the outcomes. Influences of PA on the associations between BMI and incident outcomes were evaluated. A total of 84,022 participants (mean age 50.15 years, SD 11.69; 80.3% male) were included. Over a median follow-up of 11.83 years (IQR 8.87–13.04), 3236 participants developed the primary outcome. After multivariable adjustment, a higher body mass index (BMI) and a higher waist circumference (WC) were associated with increased risks of conduction disease, but more PA was associated with a lower risk. For obese patients defined by BMI with low PA, the risk of conduction disease was higher than that of obese patients with high PA (HR: 1.42, CI: 1.21-1.66 vs. HR: 1.16, CI: 1.03–1.31). For central obese patients defined by WC with low PA, the risk of conduction disease was also higher compared to central obese patients with high PA (HR: 1.31, CI: 1.17–1.48 vs. HR: 1.12, CI: 1.03–1.23). Besides, compared to obesity with high PA, obesity with low PA was associated with a higher risk of atrioventricular block (HR: 1.70, CI: 1.28-2.27 vs. HR: 1.45, CI: 1.16-1.81) and intraventricular block (HR: 1.37, CI: 1.13-1.65 vs. HR: 1.03, CI: 0.92–1.15). Higher PA can reduce the risks of developing cardiac conduction disease, both in the obese and non-obese groups. (**Clinical Trial Registration** URL: https://www.chictr.org. Unique identifier: ChiCTRTNC-11001489).

## Introduction

Cardiac conduction disease is a common type of arrhythmia, primarily caused by tissue fibrosis that disrupts impulse initiation and propagation. In severe cases, it can become life-threatening^1,2^. Recent data consistently suggest PR interval prolongation, first-degree atrioventricular block (AVB), or bundle branch block (BBB) are associated with an increased risk of cardiovascular adverse events, including heart failure and all-cause mortality^3,4^. Pacemaker therapy is an effective solution to end-stage of conduction disease, but pacemaker-related complications and adverse results are still challenging^1^. The management of modifiable risk factors for conduction disease has become a topic of interest^5–7^.

Obesity has become a worldwide epidemic linked to poor health outcomes, and it is estimated more than half of the world’s population will be obese by 2030^8^. While numerous studies have investigated the effects of obesity on tachyarrhythmia^9,10^, there are limited data on its relationship with bradyarrhythmias. In older adults, higher body mass index (BMI) may be associated with higher risks of cardiac conduction disease, but higher levels of physical activity (PA) may reduce this risk^11^. However, there is limited research exploring how PA influences the occurrence of cardiac conduction disease in the general obese population. Additionally, BMI may not be a precise indicator of body fat distribution compared to waist circumference (WC), and little is known about the impact of WC levels on conduction disease^12^.

To address these gaps in our knowledge, this population-based prospective cohort study aimed to assess how PA influences the risk of various types of cardiac conduction disorders among individuals with different BMI categories, including AVB and intraventricular block (IVB).

## Methods

### Study population

The participants were enrolled from Kailuan Study, which is an ongoing prospective cohort study based in a community in Tangshan City, Northern China. Detailed information about the study’s design and participant group has been previously described elsewhere^13,14^. The participants were followed up with repeated questionnaires, clinical and laboratory examinations every 2 years. Participants who received questionnaires by interviews and the first survey in the 11 hospitals in 2006 were included. The exclusion criteria included participants with missing data on height, weight, electrocardiogram (ECG) records, physical exercise, and occupational information; those with a history of cardiac conduction disease in 2006; individuals with medical history involving atrial fibrillation, myocardial infarction, and heart failure; BMI <18.5 kg/m^2^; and those with missing ECG data during the follow-up period. The protocol for this study was in accordance with the guidelines of the Helsinki Declaration and this study was approved by the Ethics Committee at the Kailuan General Hospital. Written informed consent was obtained from all the participants.

### Data collection and definition

Demographic information, medical history, and lifestyle data were collected through structured questionnaires and clinical examinations both at the baseline and during follow-up. Trained physicians conducted measurements of standing height, weight, WC, and blood pressure using standardized instruments and following a prescribed protocol. Participants wore light clothing and stood barefoot with relaxed arms. Height measurements are accurate to 0.1 cm, and weight measurements are accurate to 0.1 kg. BMI was calculated as weight (in kilograms) divided by height (in meters) squared (kg/m^2^). WC was defined as the smallest perimeter located between the last rib and the iliac crest, rounded to the nearest inch. Blood pressure was measured in the seated position using a mercury sphygmomanometer, and average levels of systolic and diastolic blood pressure were then calculated as the mean of three measurements. Fasting blood samples were collected and were measured using the Hitachi 747 auto-analyzer (Hitachi, Tokyo, Japan). Serum total cholesterol (TC), triglycerides (TG), high-density lipoprotein cholesterol (HDL-C), low-density lipoprotein cholesterol (LDL-C), fast blood glucose (FBG), and hs-CRP (high-sensitivity C reactive protein) were also measured via a standardized protocol. The estimated glomerular filtration rate (eGFR) was calculated using the Chronic Kidney Disease Epidemiology Collaboration creatinine equation.

BMI was categorized according to the recommendations of the Working Group on Obesity in China: normal-weight (18.5 ≤ BMI < 24 kg/m^2^), overweight (BMI 24 ≤ BMI < 28 kg/m^2^), and obesity (BMI ≥28 kg/m^2^)^15^. Central obesity was defined as WC ≥90 cm for men and ≥80 cm for women^16^. High PA participants were defined as participants who engaged in aerobic exercise ≥3 times/week for ≥30 min/session (e.g., walking, jogging, ball sports, swimming, etc.)^17^, according to self-reported frequency of PA from questionnaires. Furthermore, workers with an average PA of ≥1746 kcal during working days were categorized as high PA (according to the Chinese Classification Standard of Physical Labor Intensity GB3869-1997).

Adverse events (e.g., myocardial infarction, heart failure, et al) were identified following the International Classification of Diseases, Tenth Revision (ICD-10) codes. Diabetes was defined as either FBG ≥7.0 mmol/L, or self-report use of antidiabetic medications. Hypertension was defined as either blood pressure >140 mmHg or diastolic blood pressure >90 mmHg, or self-report use of antihypertensive medications. Current drinkers was defined as consuming alcoholic beverages ≥100 ml once a day on average in the past year. Smoking was defined as smoking ≥1 cigarette once a day on average in the past year. High salt intake was defined as a daily intake of >6 grams of salt.

### Outcome and follow-up

The primary outcome of the study was newly diagnosed cardiac conduction disease. The secondary outcomes were newly diagnosed AVB and IVB. Standard 12-lead ECG examinations were performed in the initial survey and subsequent biennial follow-ups. All participants underwent a 10-second 12-lead ECG, collected between 7:00 and 9:00 in the morning. The ECG measurements and diagnoses were made by at least two trained ECG physicians. Cardiac conduction disease was diagnosed as the first occurrence of any type of cardiac conduction disorder, including AVB, complete right bundle branch block (CRBBB), incomplete right bundle branch block (iRBBB), complete left bundle branch block (CLBBB), incomplete left bundle branch block (iLBBB), left anterior fascicular block (LAFB), left posterior fascicular block (LPFB), and nonspecific intraventricular conduction delay (NS-IVCD). AVB is defined as first-degree, second-degree, or third-degree AVB, and IVB includes CRBBB, iRBBB, CLBBB, iLBBB, LAFB, LPFB, or NS-IVCD. All participants were followed up from the baseline examination every two years until the occurrence of outcomes on December 31, 2019. Diagnosis information was obtained from family reports and medical records from medical insurance or hospitals during the follow-up period conducted at all 11 Kailuan hospitals. The specific ECG abnormalities were categorized using the Minnesota-coded criteria^18^, and the diagnosis of conduction disease was based on the guidelines of the ACC/AHA/HRS^14^.

### Statistical analysis

Continuous variables were described as mean ± standard deviation (SD) or median with interquartile range (IQR), and categorical variables were shown as frequencies and percentages. Missing data of covariates (missing rate <1%, Supplementary Table 1) were inputted with multiple imputations by chained equations to minimize the potential inferential. The multivariable Cox proportional hazards models were performed to analyze the association between BMI, WC, and the risk of outcomes. Cox regression was used to assess the association between BMI, WC, and the risk of outcomes based on PA levels, with the normal-weight group serving as the reference. To assess the interaction effect between BMI and PA, both additive and multiplicative interactions were analyzed. Restricted cubic spline models were utilized to explore the patterns of the association between BMI and incident outcomes. Three knots were placed at the 10th, 50th, and 90th percentiles, with the median of BMI used as the reference point. In the subsequent analysis, we further utilized the Cox models to investigate the influences of PA on the relationship between BMI, WC, and outcomes, and the normal-weight group with high PA was set as the reference group. The results are presented as hazard ratios (HR) with 95% confidence intervals (CI), and we also calculated the incidence rate of incident outcomes per 10,000 person-years.

To adjust for potential covariates, 6 models were developed. Model 1 was adjusted for age and gender. Model 2 was further adjusted for HDL-C, LDL-C, hs-CRP, eGFR, salt intake, snore, smoking status, alcohol status, hypertension, and diabetes. Model 3 was further adjusted for the use of antihypertensive, antidiabetic, and lipid-lowering medications. Model 4 and Model 5 were further adjusted for PA on the basis of Model 2 and Model 3 respectively. Model 6 and Model 7 further adjusted for BMI on the basis of Model 2 and Model 3 respectively. Kaplan–Meier curves of the cumulative incidence of outcomes were drawn stratified by different PA and BMI groups, with the difference between groups compared by the log-rank test.

Given that medical history may be associated with the risk of outcomes, subgroup analyses by diabetes and hypertension status were performed. To test the robustness and consistency of the results, sensitivity analyses were conducted. First, we excluded outcome events that occurred within less than two years of follow-up to minimize the potential for reverse causation. Second, we excluded participants developing myocardial infarction during follow-up. Third, a Fine-Gray model was used whereby all-cause mortality was used as a competing risk. Cox models were used to investigate the influences of PA on the relationship between BMI and outcomes, and the normal-weight group with high PA was set as the reference group. All analyses were performed using SAS version 9.4 and R version 4.1. All the statistical tests were 2-sided, and *P* < 0.05 was considered statistically significant.

## Results

### Study population

**Fig. 1:**
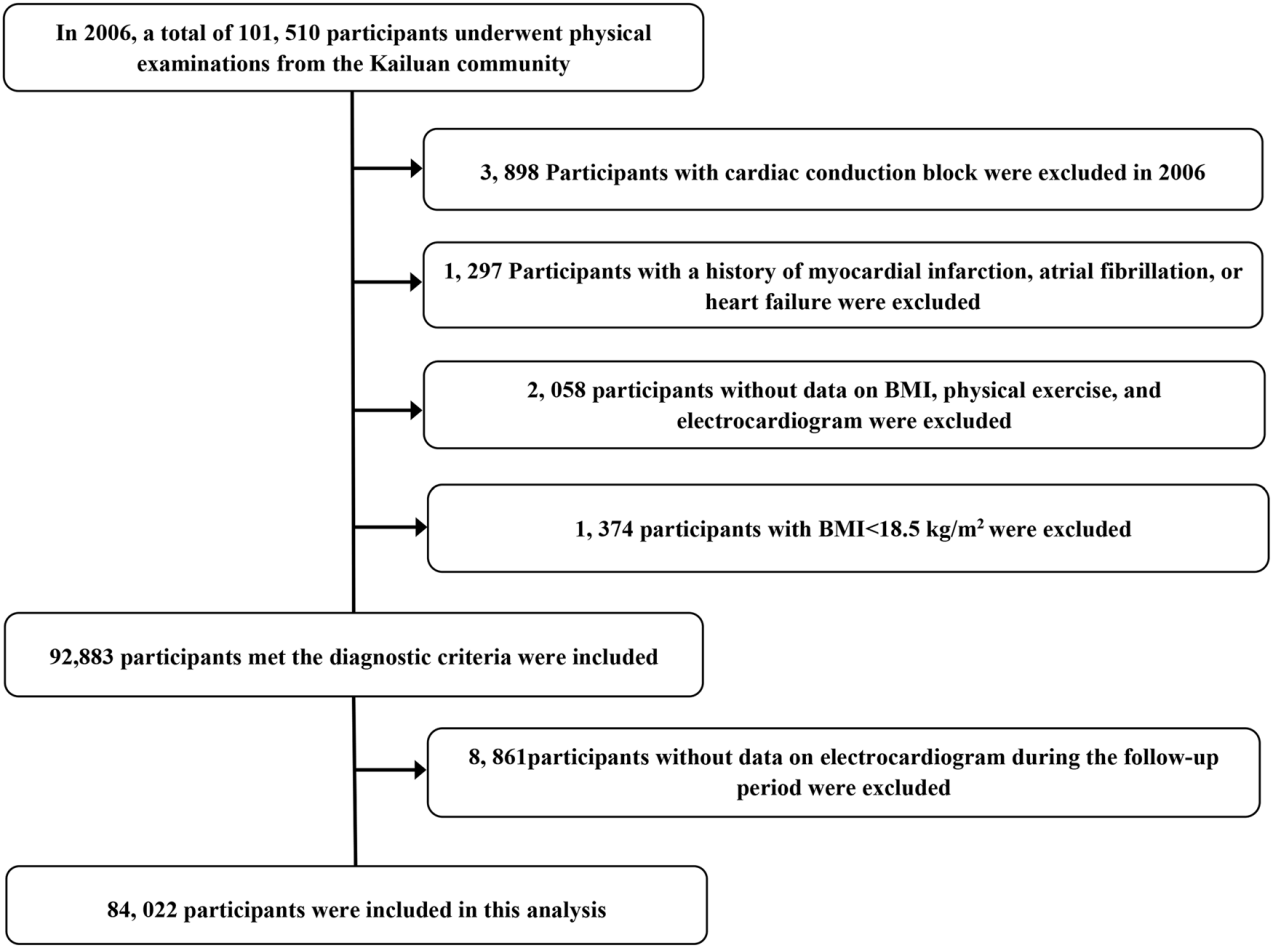
Flowchart of the study.

In 2006, a total of 101,510 participants from the Kailuan community underwent physical examinations and completed questionnaires through interviews in 11 hospitals. Among them, 92,883 participants met the diagnostic criteria and were included in the study. After excluding individuals without electrocardiographic data during the follow-up period, a total of 84,022 eligible subjects were ultimately included in this study (Fig. 1).

The baseline characteristics of all subjects based on different BMI groups are shown in Table 1, and the baseline characteristics based on different PA groups are presented in Supplementary Table 2. Of these, 80.3% were men and the mean age at the beginning of the follow-up was 50.15 ± 11.69 years. Compared with normal-weight and overweight groups, the patients with obesity were more likely to have higher levels of LDL-C, TG, TC, hs-CRP, and eGFR, lower levels of HDL-C, and a higher prevalence of hypertension and diabetes. In comparison to low PA subjects, those with high PA were more likely to be younger, male, smokers, and drinkers. They had higher levels of WC, LDL-C, TG, TC, and eGFR, lower levels of HDL-C, hs-CRP, and a lower prevalence of diabetes.

**Table 1:** Baseline characteristics of the participants based on different BMI groups

	Total (*N* = 840, 22)	18.5 ≤ BMI < 24 (*N* = 32, 449)	24 ≤ BMI < 28 (*N* = 35, 812)	BMI ≥ 28 (*N* = 15, 761)	*P*^a^
Age, year	50.15 ± 11.69	49.44 ± 12.38	50.82 ± 11.09	50.12 ± 11.47	<0.01
Male, *N* (%)	67448 (80.3)	24856 (76.6)	29762 (83.1)	12830 (81.4)	<0.01
BMI, kg/m^2^	25.17 ± 3.36	21.96 ± 1.41	25.85 ± 1.13	30.25 ± 2.24	<0.01
WC, cm	86.93 ± 9.81	80.91 ± 8.45	88.52 ± 7.53	95.75 ± 8.87	<0.01
HDL-C, mmol/L	1.54 ± 0.39	1.59 ± 0.40	1.52 ± 0.38	1.49 ± 0.38	<0.01
LDL-C, mmol/L	2.36 ± 0.86	2.28 ± 0.85	2.40 ± 0.86	2.43 ± 0.87	<0.01
TG, mmol/L	1.28 (0.90-1.95)	1.05 (0.75-1.50)	1.38 (0.99-2.08)	1.67 (1.17-2.52)	<0.01
TC, mmol/L	4.96 ± 1.15	4.86 ± 1.10	5.00 ± 1.17	5.05 ± 1.17	<0.01
hs-CRP, mg/L	0.80 (0.30-2.02)	0.56 (0.20-1.58)	0.82 (0.33-2.03)	1.20 (0.52-2.80)	<0.01
eGFR, ml/min/1.73 m^2^	83.16 ± 22.48	85.40 ± 22.23	82.08 ± 22.57	81.01 ± 22.40	<0.01
High salt intake, *N* (%)	9078 (10.8)	3128 (9.64)	3987 (11.1)	1963 (12.5)	<0.01
Snore, *N* (%)					<0.01
Never	52817 (62.9)	22255 (68.6)	21882 (61.1)	8680.0 (55.1)	
Sometimes	19422 (23.1)	7040.0 (21.7)	8665.0 (24.2)	3717.0 (23.6)	
Always	11744 (14.0)	3133.0 (9.66)	5251.0 (14.7)	3360.0 (21.3)	
Physical activity, *N* (%)	55645 (66.7)	20522 (63.7)	24462 (68.8)	10661 (68.1)	<0.01
Current smoker, *N* (%)	29096 (34.6)	11371 (35.0)	12529 (35.0)	5196 (33.0)	<0.01
Current drinker, *N* (%)	2497 (2.97)	959 (2.96)	1072 (2.99)	466 (2.96)	0.95
Hypertension, *N* (%)	34235 (41.0)	9382 (29.1)	15879 (44.6)	8974 (57.4)	<0.01
Diabetes, *N* (%)	7396 (8.87)	1800 (5.59)	3554 (9.99)	2042 (13.1)	<0.01
Antihypertension medications, *N* (%)	3144.0 (3.77)	731.00 (2.27)	1476.0 (4.15)	937.00 (5.99)	<0.01
Antidiabetic medications, *N* (%)	1839.0 (2.20)	458.00 (1.42)	886.00 (2.49)	495.00 (3.16)	<0.01
Lipid-lowering medications, *N* (%)	692.00 (0.83)	158.00 (0.49)	316.00 (0.89)	218.00 (1.39)	<0.01

*BMI* body mass index, *WC* waist circumference, *HDL-C* high-density lipoprotein cholesterol, *LDL-C* low-density lipoprotein cholesterol, *TG* triglycerides, *TC* total cholesterol, *hs-CRP* high-sensitivity C reactive protein, *eGFR* estimated glomerular filtration rate.

^a^*P*, comparison of baseline characteristics between BMI groups.

### Associations between BMI groups and outcomes

**Fig. 2:**
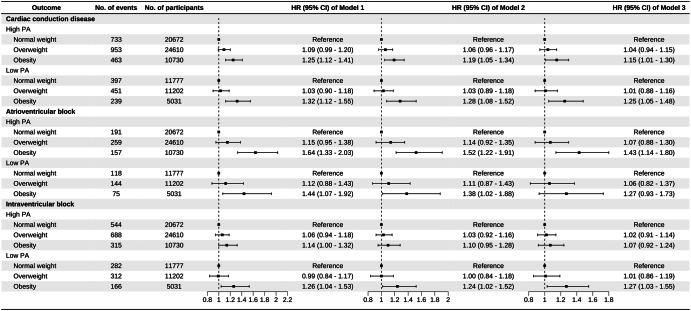
Association between BMI and the incident outcomes based on different physical activity levels. The model 1 was adjusted for age and gender; Model 2 was further adjusted for HDL-C, LDL-C, hs-CRP, eGFR, salt intake, snore, smoking status, alcohol status, hypertension, and diabetes; Model 3 was further adjusted for antihypertensive, antidiabetic, and lipid-lowering medications. HR hazard ratio, CI confidence interval, PA physical activity, BMI body mass index.

**Fig. 3:**
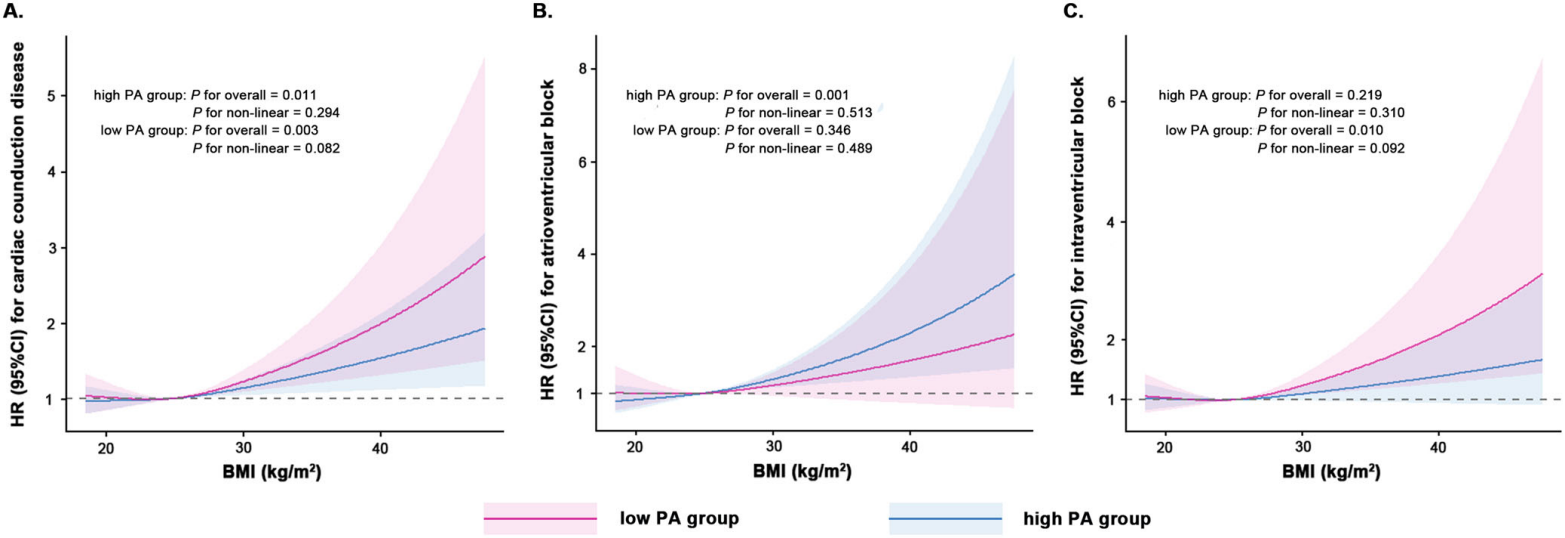
The associations between BMI and PA with the risk of cardiac conduction disease. The associations between BMI and PA with risk of **A** cardiac conduction disease, **B** atrioventricular block, and **C** intraventricular block. Cox models with restricted cubic splines were fitted to the data with 3 knots at the 10th, 50th, and 90th percentiles of the BMI. Solid lines indicate hazard ratios and dashed lines indicate 95% CIs from restricted cubic spline analysis. Pink and blue lines represented the low PA group and high PA group, respectively. HR hazard ratio, CI confidence interval, PA physical activity, BMI body mass index.

**Fig. 4:**
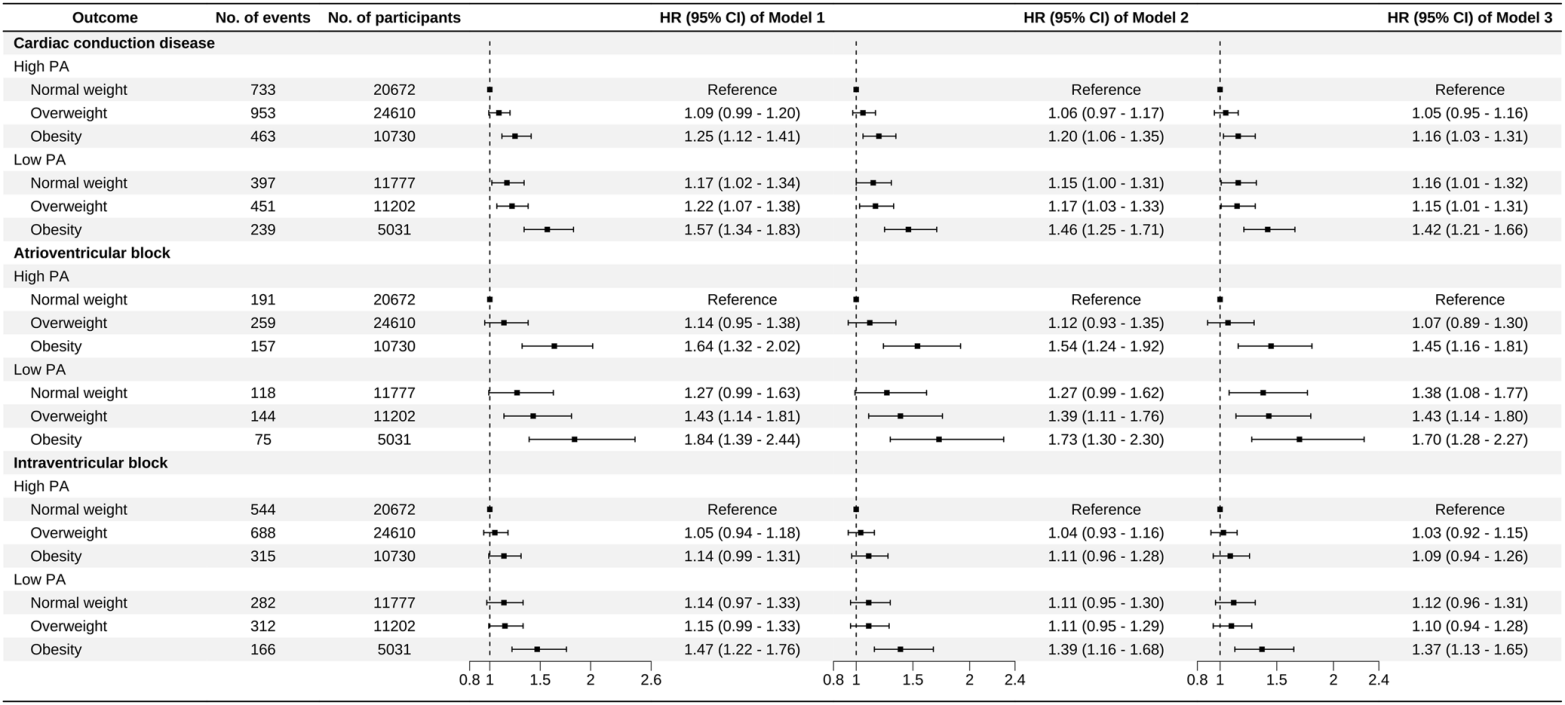
Influences of physical activity on the association between BMI and incident outcomes. The model 1 was adjusted for age and gender; Model 2 was further adjusted for HDL-C, LDL-C, hs-CRP, eGFR, salt intake, snore, smoking status, alcohol status, hypertension, and diabetes; Model 3 was further adjusted for antihypertensive, antidiabetic, and lipid-lowering medications. HR hazard ratio, CI confidence interval, PA physical activity, BMI body mass index.

**Fig. 5:**
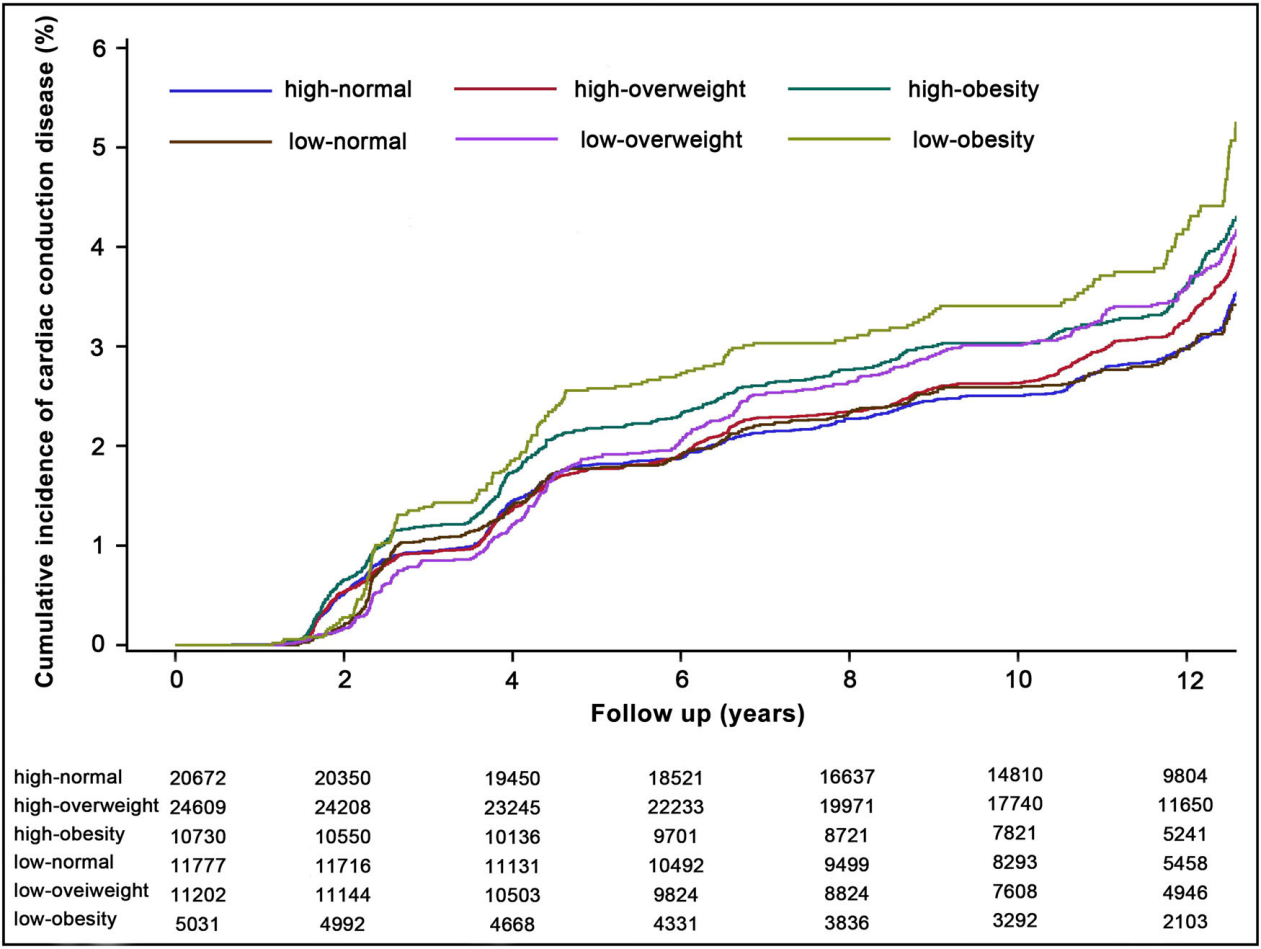
Kaplan–Meier curves of incident cardiac conduction disease according to the influences of physical activity on different BMI groups. The log-rank test compared survival distributions between groups, and statistical differences were considered (log-rank *P* < 0.01). Different groups were shown by different colored lines. High-normal was defined as a normal-weight group with high PA; high-overweight was defined as an overweight group with high PA; high-obesity was defined as an obesity group with high PA; low-normal was defined as a normal-weight group with low PA; low-overweight was defined as an overweight group with low PA; low-obesity was defined as obesity group with low PA. BMI body mass index, PA physical activity.

Over a median follow-up of 11.83 (8.87–13.04) years, we identified 3236 (3.85%) cases of incident cardiac conduction disease, including 944 (1.12%) cases of incident AVB and 2307 (2.75%) cases of incident IVB. Regarding the primary outcome, the incidence rate of cardiac conduction disease increased from 32.87 per 10,000 person-year in the normal-weight group to 42.37 per 10,000 person-year in the obesity group. As for the secondary outcomes, the incidence rates of AVB and IVB in the obesity group were 13.83 and 28.85 per 10,000 person-year, respectively. The positive associations were observed between the BMI and the risk of incident conduction disease outcomes. Compared to individuals in the normal-weight group, those in the obesity group had HRs (95% CIs) of 1.18 (1.07–1.31) for cardiac conduction disease, 1.37 (1.14–1.65) for AVB, and 1.13 (1.01–1.28) for IVB, after adjusting for covariates in Model 3 (Table 2). Furthermore, individuals with low PA had a higher risk of outcomes compared to those with high PA. When different BMI groups were stratified based on PA levels, the association between obesity and the risk of incident conduction disease development remained robust (Fig. 2). No interaction was observed between PA and BMI. The confidence intervals for the relative excess risk due to interaction and attributable proportion included 0, while the confidence interval for the synergy index included 1 and the *P* value for interaction was >0.05. The restricted cubic spline curves indicated linear positive relationships between BMI and risk of cardiac conduction disease (*P*_overall association_ < 0.05 and *P*_non-linear association_ > 0.05, respectively). Additionally, there was no association observed between BMI and PA and the risk of incident AVB in the low PA group and IVB in the high PA group (*P*_overall association_ > 0.05 and *P*_non-linear association_ > 0.05, respectively) (Fig. 3). For obese patients with low PA, the incidence rate of conduction disease was higher than that of obese patients with high PA (HR: 1.42, CI: 1.21-1.66 vs. HR: 1.16, CI: 1.03–1.31) (Fig. 4). Among different outcomes, AVB was related to the highest incidence for obese patients with low PA compared to normal-weight participants with high PA (HR: 1.70, CI: 1.28-2.27). As shown in Fig. 5 and Supplementary Figs. 1, 2, participants in the obesity with low PA group experienced a higher risk of outcomes than participants of other groups during the follow-up period (*P* < 0.01 for log-rank).

**Table 2:** Association of BMI and physical activity with the incident outcomes

	Cardiac conduction disease			Atrioventricular block			Intraventricular block		
	Case/total	Incidence rate^a^	HR(95% CI)	Case/total	Incidence rate^a^	HR(95% CI)	Case/total	Incidence rate^a^	HR(95% CI)
**BMI**									
Model 1^b^									
Normal-weight group	1130/32449	32.87	Reference	309/32449	8.89	Reference	826/32449	24.00	Reference
Overweight group	1404/35812	37.12	1.07 (0.99–1.16)	403/35812	10.53	1.14 (0.98-1.32)	1000/35812	26.33	1.04 (0.95–1.14)
Obesity group	702/15761	42.37	1.28 (1.17–1.41)	232/15761	13.83	1.57 (1.33–1.86)	481/15761	28.85	1.19 (1.06–1.33)
Model 4^c^									
Normal-weight group	1130/32449	32.87	Reference	309/32449	8.89	Reference	826/32449	24.00	Reference
Overweight group	1404/35812	37.12	1.05 (0.97–1.13)	403/35812	10.53	1.11 (0.95–1.29)	1000/35812	26.33	1.02 (0.93–1.13)
Obesity group	702/15761	42.37	1.22 (1.11–1.35)	232/15761	13.83	1.48 (1.23–1.77)	481/15761	28.85	1.16 (1.02–1.30)
Model 5^d^									
Normal-weight group	1130/32449	32.87	Reference	309/32449	8.89	Reference	826/32449	24.00	Reference
Overweight group	1404/35812	37.12	1.03(0.95–1.12)	403/35812	10.53	1.06 (0.91–1.23)	1000/35812	26.33	1.01 (0.92–1.11)
Obesity group	702/15761	42.37	1.18 (1.07–1.31)	232/15761	13.83	1.37 (1.14–1.65)	481/15761	28.85	1.13 (1.01–1.28)
**Physical activity**									
Model 1^b^									
High PA	2149/56012	36.16	Reference	607/56012	10.09	Reference	1547/56012	25.92	Reference
Low PA	1087/28010	37.05	1.17 (1.07–1.27)	337/28010	11.36	1.23 (1.05–1.44)	760/28010	25.79	1.15 (1.03–1.27)
Model 6^e^									
High PA	2149/56012	36.16	Reference	607/56012	10.09	Reference	1547/56012	25.92	Reference
Low PA	1087/28010	37.05	1.14 (1.04–1.24)	337/28010	11.36	1.22 (1.04–1.43)	760/28010	25.79	1.12 (1.01–1.24)
Model 7^f^									
High PA	2149/56012	36.16	Reference	607/56012	10.09	Reference	1547/56012	25.92	Reference
Low PA	1087/28010	37.05	1.14 (1.05–1.25)	337/28010	11.36	1.23 (1.05–1.45)	760/28010	25.79	1.12 (1.01–1.25)

*BMI* body mass index, *HR* hazard ratio, *CI* confidence interval, *PA* physical activity.

^a^Per 10,000 person-years;

^b^The model was adjusted for age and gender；

^c^The model was further adjusted for HDL-C, LDL-C, hs-CRP, eGFR, salt intake, snore, smoking status, alcohol status, hypertension, diabetes, and physical activity on the basis of Model 1;

^d^The model was further adjusted for antihypertensive, antidiabetic, and lipid-lowering medications on the basis of Model 4;

^e^The model was further adjusted for HDL-C, LDL-C, hs-CRP, eGFR, salt intake, snore, smoking status, alcohol status, hypertension, diabetes, and BMI on the basis of Model 1;

^f^The model was further adjusted for antihypertensive, antidiabetic, and lipid-lowering medications on the basis of Model 6.

### Association between WC groups and outcomes

Compared to the normal-WC group, the central obesity group showed a higher risk of the primary outcome (HR: 1.14, 95% CI: 1.06–1.23), as well as the secondary outcomes of AVB (HR: 1.19, 95% CI: 1.04–1.36) and IVB (HR: 1.12; 95% CI: 1.03–1.22) (Supplementary Table 3). When WC groups were stratified according to PA levels, the results remained consistent (Supplementary Table 4). In central obese patients with low PA, the risk of conduction disease was higher than in obesity with high PA (HR: 1.31, CI: 1.17–1.48 vs. HR: 1.12, CI: 1.03–1.23) (Supplementary Table 5). AVB was related to the highest incidence for central obese patients with low PA compared to normal-WC participants with high PA (HR: 1.42, CI: 1.15–1.76).

### Subgroup analysis

The subgroup analyses by hypertension and diabetes were presented in Supplementary Table 6. Among hypertension patients with obesity, the patients with low PA have a 28% higher risk of conduction disease compared to patients with high PA (HR: 1.42, CI: 1.14–1.77 vs. HR: 1.14, CI: 0.95–1.37). For diabetes patients, we found obese patients with low PA have a 57% higher risk of conduction disease compared to obese patients with high PA (HR: 1.94, CI: 1.19–3.17 vs. HR: 1.37, CI: 0.88–2.12).

### Sensitivity analysis

The results of sensitivity analyses, as shown in Supplementary Table 7, generally supported the findings of the main analyses. In this study, after excluding 1062 outcomes that occurred within the first 2 years of follow-up, the higher risk of incident outcomes remained associated with obesity with low PA compared to obesity with high PA. Similar results were obtained when we excluded 1120 participants who developed myocardial infarction during follow-up. A total of 7704 mortality cases were identified, and the competing risk of mortality did not apparently influence the stability of the association.

## Discussion

In this prospective cohort study, obesity (BMI ≥28 kg/m^2^) and low PA were associated with an increased risk of cardiac conduction disease. Even when PA levels were similar, obese individuals still had a higher prevalence of cardiac conduction disease. Secondly, among obese patients, those with high PA may be related to have a reduced risk of developing conduction disease compared to those with low PA, demonstrating PA has a protective effect. These consistent findings were observed in individuals with central obesity, defined by WC.

To the best of our knowledge, this is the first study to investigate the association between PA and incident conduction disease based on different BMI and WC categories, expanding on previous research that predominantly focused on single conduction abnormalities or specific demographics, such as men or the elderly^2,5,11,19^. Indeed, the relationships between increased PA and reduced risks of cardiovascular disease and all-cause mortality are well-established^20^. In this study, we also found that high PA potentially mitigates the risk of cardiac conduction diseases in obese patients. This could be attributed to a combination of indirect and direct effects. Indirectly, PA could reduce the risk of conduction disease by mediating risk factors, including lowering the risk of obesity and weight gain and assisting in the management of coronary heart disease and diabetes mellitus^21–24^. In addition, some direct impacts of PA may also lower the risk of conduction disease by improving endothelial dysfunction, improving cardiac function, reducing interstitial fibrosis, and decreasing systemic inflammation^25–27^. Given that cardiac conduction disease arises from fibrosis and fibrosis may be related to an initial inflammatory process^28^, this offers a potential explanation for the observed protective effect of increased PA against the development of conduction disease. The data implies that effectively promoting PA could potentially reduce a significant portion of conduction disease in the general population. When it comes to tachyarrhythmia, weight reduction has been shown to be effective in preventing atrial fibrillation recurrences and alleviating symptoms in obese patients^29^. Nevertheless, PA might increase atrial fibrillation risk when individuals engage in frequent exercise and with high intensity, especially for vigorous exercise in younger adulthood and elite athletes^30^.

Body fat distribution is an important risk factor for obesity-related diseases. WC measurement offers a cost-effective alternative to expensive imaging techniques^31,32^. Central obesity, as measured by WC, has previously been shown to provide valuable additional predictive information for major metabolic issues, including myocardial infarction^33^. Furthermore, WC trajectories have been demonstrated to be independent of BMI when predicting the risk of developing cardiovascular disease, suggesting that WC may be a more precise predictor than BMI^34^. Zhao et al. has reported that elevated levels of WC were positively associated with the risk of atrial fibrillation^35^. Our current study found that central obesity is associated with an increased risk of incident conduction disease, and the results are robust across different PA subgroups. This study represents one of the first attempts to assess the impact of central obesity on the development of conduction disease. Additionally, we observed that PA plays a protective role in improving incident outcomes among central obese patients. In terms of the association between BMI and body composition, an individual with a high BMI and high PA may have a higher muscle mass, whereas an individual with a high BMI and low PA may present a higher fat component, indicating an increased fat component and its complications may be associated with the conduction disease outcome^36^.

The study revealed slightly different effects of obesity on various conduction diseases. However, it is important to note that due to limited research in this field, explaining the results for various conduction disease sites can be challenging. While recent literature has extensively explored the risk factors for AVB^5,37^, there remains limited findings on those of IVB. Obesity is associated with an increased risk of diabetes and hypertension. Kerola et al. found fasting glucose levels and suboptimal blood pressure are related to AVB^5^. Diabetes leads to various metabolic changes in cardiomyocytes which can contribute to cardiomyocyte injury and cell death, subsequently triggering inflammation and fibrosis^38^. In the high PA group, the increase in the risk of atrioventricular block is greater than that in the low PA group, which may be associated with a higher incidence of first-degree atrioventricular block in the active PA population^39^.

We observed that compared to the general obese individuals, obese patients with diabetes may benefit more from PA. Besides, it is well-known hypertension and associated left ventricular hypertrophy often coexist with myocardial fibrosis^40^. The potential mechanism might be elevated blood pressure, which can lead to local fibrosis infiltrating the AV conduction system^41^. Therefore, it may be worthwhile to control blood pressure effectively to prevent conduction disease and AVB. In the case of IVB, conduction disturbances of electrical signals may be associated with degenerative changes, heart structural dysfunction, or other etiologies^1^. The mechanisms potentially linking LBBB to hypertension include increased ventricular pressure load and ventricular remodeling^42^.

The Cardiovascular Health Study of 5050 participants explored the connections between lifestyle habits and conduction^11^. In that study, older age, male sex, a larger BMI, and diabetes were found to be associated with incident conduction disease. Amongst lifestyle habits, a higher level of PA was linked to a decreased risk, although smoking and alcohol consumption did not exhibit significant associations. Our study further confirmed the protective role of PA, especially in obese individuals, suggesting that daily necessary exercise could be a crucial factor in managing cardiac conduction disease risks, regardless of weight loss achievements.

Our study has several major strengths. The study is the first large community-based study with long follow-up period to evaluate the association between obesity, central obesity and cardiac conduction disease. The selection of study population may contribute to avoiding selection bias, and comprehensive information were prospectively collected according to predefined protocols. Multiple subgroup analyses and sensitivity analyses were further conducted to validate the findings, yielding consistent results.

It is important to acknowledge several limitations. Firstly, the nature of the observational study design prevents us from identifying causal relationships between obesity and the risks of cardiac conduction. Although the findings might represent effects on overall cardiovascular events rather than direct consequences on the conduction system, individuals with baseline atrial fibrillation, myocardial infarction, and heart failure were excluded. Besides, our model has adjusted for most demographic and clinical covariates, but some residual or unmeasured confounders could exist. Secondly, the covariates were ascertained from self-reported questionnaires, which might induce recall bias. Thirdly, we did not identify every possible cause of conduction disease or low PA. Comorbidities associated with our outcome of interest could potentially result in a decrease in the accuracy of the results. Fourthly, most conduction diseases are inherently concealed and may be misdiagnosed through 12-lead ECGs. Consequently, the prevalence of conduction diseases could be substantially underestimated. Finally, all participants were included from the Kailuan cohort, so whether our conclusions could be generalized to other populations should be further tested.

In conclusion, higher BMI and larger WC are associated with an increased risk of incident cardiac conduction disease. Performing more PA may experience less cardiac conduction disease, and obese patients with high PA may be related to having a lower risk compared to obese patients with low PA. Such modifiable lifestyle behaviors could assist in reducing invasive interventions and optimizing cardiovascular management for obese patients.

## Supplementary Information

Supplementary Information

## Data Availability

The datasets used and/or analyzed during the current study available from the corresponding author on reasonable request.
