# Supplementary material for: Obesity and physical inactivity are associated with increased risks of cardiac conduction disease: a report from the Kailuan Cohort Study
Source: NPJ Cardiovasc Health. 2024 Jul 25;1:9. doi: 10.1038/s44325-024-00008-8 (PMC12912420; doi:10.1038/s44325-024-00008-8)
Supplement: Supplementary file 1 — Supplementary Information [file 44325_2024_8_MOESM1_ESM.pdf]

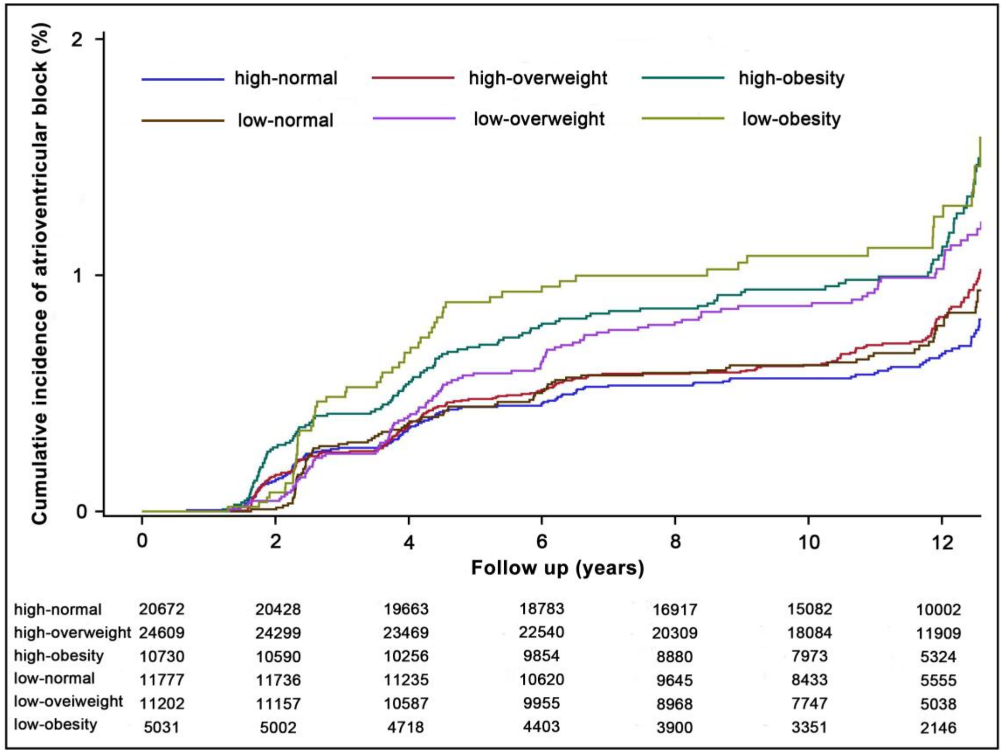

Supplementary figure 1. Kaplan-Meier curves of incident atrioventricular block according to the influences of physical activity on different BMI groups.

The log-rank test compared survival distributions between groups, and statistical differences were considered (log-rank P<0.01). Different groups were shown by different colored lines. High-normal was defined as normal-weight group with high PA; high-overweight was defined as overweight group with high PA; high-obesity was defined as obesity group with high PA; low-normal was defined as normal-weight group with low PA; low-overweight was defined as overweight group with low PA; low-obesity was defined as obesity group with low PA. BMI, body mass index; PA, physical activity.

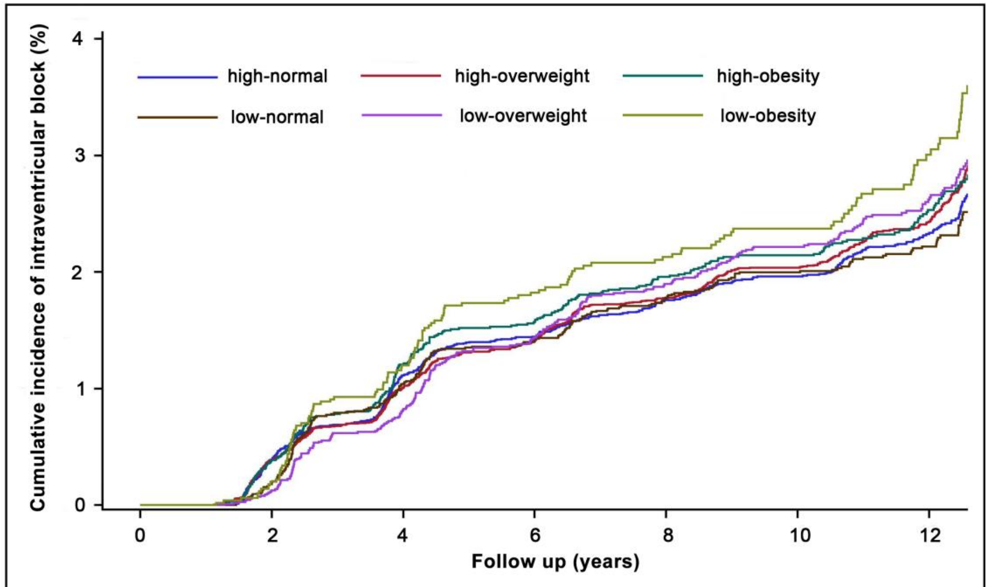

high-normal	20672	20376	19515	18603	16721	14891	9863
high-overweight	24609	24245	23326	22345	20086	17851	11744
high-obesity	10730	10578	10190	9774	8795	7895	5268
low-normal	11777	11717	11171	10540	9550	8340	5488
low-oveiweight	11202	11148	10541	9882	8886	7658	4983
low-obesity	5031	4996	4699	4372	3875	3329	2121

Supplementary figure 2. Kaplan-Meier curves of incident intraventricular block according to the influences of physical activity on different BMI groups.

The log-rank test compared survival distributions between groups, and statistical differences were considered (log-rank $P<0.01$). Different groups were shown by different colored lines. High-normal was defined as normal-weight group with high PA; high-overweight was defined as overweight group with high PA; high-obesity was defined as obesity group with high PA; low-normal was defined as normal-weight group with low PA; low-overweight was defined as overweight group with low PA; low-obesity was defined as obesity group with low PA. BMI, body mass index; PA, physical activity.

Supplementary table 1. Missing data of baseline characteristics

Variables	No. of missing data (%)
Age	NA
Male	NA
BMI	NA
WC	27 (0.03%)
HDL-C	449 (0.53%)
LDL-C	541 (0.64%)
TG	464 (0.55%)
TC	431 (0.51%)
hs-CRP	1011 (1.20%)
eGFR	540 (0.64%)
High salt intake	637 (0.76%)
Snore	636 (0.76%)
Current smoker	627 (0.75%)
Current drinker	586 (0.70%)
Hypertension	NA
Diabetes	NA
Antihypertension medications	NA

Antidiabetic medications	NA
Lipid-lowering medications	NA

BMI, body mass index; WC, waist circumference; HDL-C, high-density lipoprotein cholesterol; LDL-C, low-density lipoprotein cholesterol; TG, triglycerides; TC, total cholesterol; hs-CRP, high-sensitivity C reactive protein; eGFR, estimated glomerular filtration rate.

Supplementary table 2. Baseline characteristics of the participants based on different physical activity groups

	**Total (N = 84, 022)**	**Low PA (N = 28, 010)**	**High PA (N=56, 012)**	***P*** [a]
Age, year	50.15±11.69	54.02±13.19	48.22±10.33	<0.01
Male, N (%)	67448(80.3)	13654(48.7)	53794(96.0)	<0.01
BMI, kg/m^2	25.17±3.36	24.98±3.51	25.27±3.28	<0.01
WC, cm	86.93±9.81	86.07±10.59	87.37±9.36	<0.01
HDL-C, mmol/L	1.54±0.39	1.58±0.40	1.52±0.38	<0.01
LDL-C, mmol/L	2.36±0.86	2.24±0.87	2.42±0.85	<0.01
TG, mmol/L	1.28(0.90-1.95)	1.22(0.86-1.82)	1.31(0.92-2.03)	<0.01
TC, mmol/L	4.96±1.15	4.92±1.12	4.98±1.16	<0.01
hs-CRP, mg/L	0.80 (0.30-2.02)	0.90(0.31-2.50)	0.72(0.30-1.82)	<0.01

eGFR, ml/min/1.73 m^2	83.16±22.48	79.51±21.22	84.99±22.87	<0.01
Snore, N (%)				<0.01
Never	52817 (62.9)	18785 (67.1)	34032 (60.8)	
Sometimes	19422 (23.1)	6054 (21.6)	13368 (23.9)	
Always	11744 (14.0)	3169 (11.3)	8575 (15.3)	
High salt intake, N (%)	9078(10.8)	2356(8.41)	6722(12.0)	<0.01
Current smoker, N (%)	29096(34.6)	4939(17.6)	24157(43.1)	<0.01
Current drinker, N (%)	2497(2.97)	402(1.44)	2095(3.74)	<0.01
Hypertension, N (%)	34485(41.0)	11019(39.3)	23466(41.9)	<0.01
Diabetes, N (%)	7440(8.85)	2644(9.44)	4796(8.56)	<0.01
Antihypertension medications, N (%)	3166(3.77)	1206(4.31)	1960(3.50)	<0.01
Antidiabetic medications, N (%)	1851(2.20)	760(2.71)	1091(1.95)	<0.01
Lipid-lowering medications, N (%)	698(0.83)	301(1.07)	397(0.71)	<0.01

[a] *P*, comparison of baseline characteristics between physical activity groups.

PA, physical activity; BMI, body mass index; WC, waist circumference; HDL-C, high-density lipoprotein cholesterol; LDL-C, low-density lipoprotein cholesterol; TG, triglycerides; TC, total cholesterol; hs-CRP, high-sensitivity C reactive protein; eGFR, estimated glomerular filtration rate.

Supplementary table 3. Association between waist circumference and the incident outcomes

	**Cardiac conduction disease**			**Atrioventricular block**			**Intraventricular block**		
	Case/total	Incidence rate [a]	HR(95%CI)	Case/total	Incidence rate [a]	HR(95%CI)	Case/total	Incidence rate [a]	HR(95%CI)
**Model 1** [b]									
Normal-WC	1179/35598	31.00	Reference	343/35598	8.93	Reference	826/32449	24.00	Reference
Central obesity	2057/48424	40.55	1.22 (1.14-1.31)	601/48424	11.70	1.35 (1.18-1.53)	1000/35812	26.33	1.17 (1.07-1.27)
**Model 4** [c]									
Normal-WC	1179/35598	31.00	Reference	343/35598	8.93	Reference	826/32449	24.00	Reference
Central obesity	2057/48424	40.55	1.17 (1.08-1.25)	601/48424	11.70	1.24 (1.09-1.42)	1000/35812	26.33	1.14 (1.04-1.24)
**Model 5** [d]									
Normal-WC	1179/35598	31.00	Reference	343/35598	8.93	Reference	826/32449	24.00	Reference
Central obesity	2057/48424	40.55	1.14 (1.06-1.23)	601/48424	11.70	1.19 (1.04-1.36)	1000/35812	26.33	1.12 (1.03-1.22)

[a] Per 10, 000 person-years;

[b] The model was adjusted for age and gender;

[c] The model was further adjusted for HDL-C, LDL-C, hs-CRP, eGFR, salt intake, snore, smoking status, alcohol status, hypertension, diabetes and physical activity on the basis of Model 1;

[d] The model was further adjusted for antihypertensive, antidiabetic and lipid-lowering medications on the basis of Model 4.

HR, hazard ratio; CI, confidence interval; WC, waist circumference.

Supplementary table 4. Association between waist circumference and the incident outcomes based on different physical activity levels

	Cardiac conduction disease			Atrioventricular block			Intraventricular block		
	Case/total	Incidence rate [a]	HR(95%CI)	Case/total	Incidence rate [a]	HR(95%CI)	Case/total	Incidence rate [a]	HR(95%CI)
**Active PA**									
Model 1 [b]									
Normal-WC	834/24250	32.20	Reference	226/24250	8.63	Reference	544/20672	24.78	Reference
Central obesity	1315/31762	39.22	1.20(1.10-1.31)	381/31762	11.22	1.41 (1.20-1.67)	688/24610	26.22	1.11 (1.00-1.23)
Model 2 [c]									
Normal-WC	834/24250	32.20	Reference	226/24250	8.63	Reference	544/20672	24.78	Reference
Central obesity	1315/31762	39.22	1.140(1.04-1.25)	381/31762	11.22	1.30 (1.10-1.54)	688/24610	26.22	1.08 (0.97-1.20)
Model 3 [d]									
Normal-WC	834/24250	32.20	Reference	226/24250	8.63	Reference	544/20672	24.78	Reference
Central obesity	1315/31762	39.22	1.11 (1.02-1.22)	381/31762	11.22	1.26 (1.07-1.49)	688/24610	26.22	1.06 (0.95-1.12)
**Inactive PA**									
Model 1 [b]									
Normal-WC	345/11348	28.42	Reference	117/11348	9.56	Reference	282/11777	22.48	Reference

Central obesity	742/16662	43.13	1.24 (1.11-1.41)	220/16662	12.63	1.22 (0.98-1.51)	312/11202	26.56	1.25 (1.09-1.45)
Model 2 [c]									
Normal-WC	345/11348	28.42	Reference	117/11348	9.56	Reference	282/11777	22.48	Reference
Central obesity	742/16662	43.13	1.20 (1.06-1.36)	220/16662	12.63	1.14 (0.91-1.42)	312/11202	26.56	1.23 (1.06-1.42)
Model 3 [d]									
Normal-WC	345/11348	28.42	Reference	117/11348	9.56	Reference	282/11777	22.48	Reference
Central obesity	742/16662	43.13	1.18 (1.04-1.33)	220/16662	12.63	1.07 (0.85-1.34)	312/11202	26.56	1.22 (1.05-1.42)

[a] Per 10, 000 person-years;

[b] The model was adjusted for age and gender;

[c] The model was further adjusted for HDL-C, LDL-C, hs-CRP, eGFR, salt intake, snore, smoking status, alcohol status, hypertension and diabetes on the basis of Model 1;

[d] The model was further adjusted for antihypertensive, antidiabetic and lipid-lowering medications on the basis of Model 2.

HR, hazard ratio; CI, confidence interval; PA, physical activity; WC, waist circumference.

Supplementary table 5. Influences of physical activity on the association between waist circumference and incident outcomes

	**Cardiac conduction disease**			**Atrioventricular block**			**Intraventricular block**		
	Case/total	Incidence rate [a]	HR(95%CI)	Case/total	Incidence rate [a]	HR(95%CI)	Case/total	Incidence rate [a]	HR(95%CI)
Model 1 [b]									
**High PA**									
Normal-WC	834/24250	32.20	Reference	226/24250	8.63	Reference	616/24250	23.70	Reference
Central obesity	1315/31762	39.22	1.20 (1.10-1.30)	381/31762	11.22	1.42 (1.21-1.66)	931/31762	27.63	1.11 (1.00-1.23)
**Low PA**									
Normal-WC	345/11348	28.42	1.13 (1.01-1.26)	117/11348	9.56	1.30 (1.07-1.60)	233/11348	19.13	1.07 (0.94-1.22)
Central obesity	742/16662	43.13	1.43 (1.27-1.60)	220/16662	12.63	1.58 (1.28-1.95)	527/16662	30.47	1.37 (1.19-1.56)
Model 2 [c]									
**High PA**									
Normal-WC	834/24250	32.20	Reference	226/24250	8.63	Reference	616/24250	23.70	Reference
Central obesity	1315/31762	39.22	1.14 (1.05-1.25)	381/31762	11.22	1.31 (1.11-1.54)	931/31762	27.63	1.08 (0.97-1.20)
**Low PA**									
Normal-WC	345/11348	28.42	1.11 (0.99-1.23)	117/11348	9.56	1.29 (1.06-1.58)	233/11348	19.13	1.05 (0.92-1.19)

Central obesity	742/16662	43.13	1.34 (1.19-1.50)	220/16662	12.63	1.47 (1.19-1.82)	527/16662	30.47	1.31 (1.14-1.50)
Model 3 [d]									
**High PA**									
Normal-WC	834/24250	32.20	Reference	226/24250	8.63	Reference	616/24250	23.70	Reference
Central obesity	1315/31762	39.22	1.12 (1.03-1.23)	381/31762	11.22	1.26 (1.07-1.48)	931/31762	27.63	1.07 (0.96-1.19)
**Low PA**									
Normal-WC	345/11348	28.42	1.11 (1.10-1.24)	117/11348	9.56	1.31 (1.07-1.60)	233/11348	19.13	1.05 (0.92-1.20)
Central obesity	742/16662	43.13	1.31 (1.17-1.48)	220/16662	12.63	1.42 (1.15-1.76)	527/16662	30.47	1.29 (1.13-1.48)

[a] Per 10, 000 person-years;

[b] The model was adjusted for age and gender;

[c] The model was further adjusted for HDL-C, LDL-C, hs-CRP, eGFR, salt intake, snore, smoking status, alcohol status, hypertension and diabetes on the basis of Model 1;

[d] The model was further adjusted for antihypertensive, antidiabetic and lipid-lowering medications on the basis of Model 2.

HR, hazard ratio; CI, confidence interval; PA, physical activity; WC, waist circumference.

Supplementary table 6. Subgroup analyses [a] of association between BMI and the incident outcomes based on different physical activity levels

	Cardiac conduction disease			Atrioventricular block			Intraventricular block		
	Case/total	Incidence rate [b]	HR(95%CI)	Case/total	Incidence rate [b]	HR(95%CI)	Case/total	Incidence rate [b]	HR(95%CI)
**Hypertension**									
High PA									
Normal-weight group	224/6279	35.01	Reference	52/6279	8.03	Reference	173/6279	26.94	Reference
Overweight group	423/11060	37.04	1.04 (0.88-1.22)	106/11060	9.16	1.09 (0.78-1.52)	313/11060	27.28	1.01 (0.83-1.21)
Obesity group	258/6127	40.16	1.14 (0.95-1.37)	87/6127	13.38	1.55 (1.09-2.21)	174/6127	26.91	1.03 (0.83-1.27)
Low PA									
Normal-weight group	144/3185	45.88	1.21 (0.97-1.51)	36/3185	11.29	1.24 (0.80-1.93)	110/3185	34.89	1.23 (0.95-1.59)
Overweight group	241/4928	49.03	1.25 (1.03-1.52)	79/4928	15.84	1.61 (1.11-2.32)	161/4928	32.56	1.14 (0.90-1.43)
Obesity group	148/2906	51.18	1.42 (1.14-1.77)	42/2906	14.33	1.53 (1.00-2.34)	106/2906	36.38	1.39 (1.08-1.80)
**Diabetes**									
High PA									
Normal-weight group	34/1154	29.17	Reference	12/1154	10.20	Reference	21/1154	17.94	Reference
Overweight group	104/2318	44.80	1.47 (0.99-2.17)	28/2318	11.89	1.13 (0.57-2.23)	76/2318	32.54	1.69 (1.04-2.75)
Obesity group	57/1324	42.31	1.37 (0.88-2.12)	21/1324	15.37	1.53 (0.74-3.20)	35/1324	25.77	1.30 (0.74-2.26)

Low PA									
Normal-weight group	14/661	21.58	0.80 (0.42-1.51)	5/661	7.65	0.80 (0.28-2.31)	9/661	13.82	0.89 (0.40-1.98)
Overweight group	47/1252	37.82	1.30 (0.82-2.06)	15/1252	11.93	1.13 (0.52-2.46)	33/1252	26.43	1.56 (0.88-2.76)
Obesity group	37/731	52.43	1.94 (1.19-3.17)	15/731	20.02	2.30 (1.10-5.11)	24/731	33.67	2.05 (1.10-3.79)

[a] The model was adjusted for age, gender, HDL-C, LDL-C, hs-CRP, eGFR, salt intake, snore, smoking status, alcohol status, hypertension, diabetes, antihypertensive, antidiabetic and lipid-lowering medications;

[b] Per 10, 000 person-years.

BMI, body mass index; HR, hazard ratio; CI, confidence interval; PA, physical activity.

Supplementary table 7. Sensitivity analyses [a] of association between BMI and the incident outcomes based on different physical activity levels

	**Cardiac conduction disease**	**Atrioventricular block**	**Intraventricular block**
		Adjusted HR (95%CI)	
**Sensitivity analysis excluding outcome events occurred within the second years of follow-up (N=1062).**			
High PA			
Normal-weight group	Reference	Reference	Reference
Overweight group	1.05 (0.94-1.16)	1.07 (0.87-1.32)	1.04 (0.92-1.17)
Obesity group	1.14 (2.00-1.30)	1.36 (1.06-1.73)	1.10 (0.94-1.28)
Low PA			
Normal-weight group	1.27 (1.10-1.46)	1.48 (1.15-1.92)	1.21 (1.04-1.43)
Overweight group	1.26 (1.11-1.44)	1.47 (1.15-1.88)	1.21 (1.04-1.42)
Obesity group	1.53 (1.30-1.81)	1.73 (1.28-2.34)	1.49 (1.23-1.82)
**Sensitivity analysis excluding the participants developing myocardial infarction during follow-up (N=1120).**			
High PA			
Normal-weight group	Reference	Reference	Reference
Overweight group	1.05 (0.96-1.16)	1.09 (0.90-1.32)	1.04 (0.92-1.16)
Obesity group	1.18 (1.05-1.34)	1.45 (1.16-1.81)	1.12 (0.97-1.29)
Low PA			
Normal-weight group	1.17 (1.02-1.34)	1.30 (1.01-1.66)	1.13 (0.96-1.32)

Overweight group	1.17 (1.03-1.33)	1.34 (1.06-1.69)	1.13 (0.97-1.31)
Obesity group	1.46 (1.25-1.71)	1.67 (1.25-2.22)	1.41 (1.17-1.70)
**Competing risk model with the all-cause mortality as a competing risk (N=7704).**			
High PA			
Normal-weight group	Reference	Reference	Reference
Overweight group	1.05 (0.96-1.16)	1.09 (0.91-1.32)	1.03 (0.92-1.16)
Obesity group	1.17 (1.04-1.32)	1.46 (1.12-1.82)	1.10 (0.95-1.27)
Low PA			
Normal-weight group	1.13 (0.99-1.30)	1.26 (0.98-1.62)	1.10 (0.94-1.28)
Overweight group	1.15 (1.01-1.30)	1.36 (1.07-1.71)	1.09 (0.94-1.27)
Obesity group	1.40 (1.19-1.63)	1.60 (1.20-2.14)	1.34 (1.11-1.62)

[a] The model was adjusted for age, gender, HDL-C, LDL-C, hs-CRP, eGFR, salt intake, snore, smoking status, alcohol status, hypertension, diabetes, antihypertensive, antidiabetic and lipid-lowering medications .

BMI, body mass index; HR, hazard ratio; CI, confidence interval; PA, physical activity.
